# Parasitoid Diversity and Biological Control of *Bactericera tremblayi* and *Bactericera trigonica* (Hemiptera: Psylloidea) in Horticultural Crops in Spain

**DOI:** 10.3390/insects17080848

**Published:** 2026-08-14

**Authors:** Yolanda Santiago-Calvo, Víctor de Paz, Saskia Bastin, Lucian Fusu, Laura Baños-Picón, Diego Flores-Pérez, M. Isabel Pozo-Romero, M. Carmen Asensio-S.-Manzanera

**Affiliations:** 1 Instituto Tecnológico Agrario de Castilla y León (ITACyL), Ctra. de Burgos Km. 119, 47071 Valladolid, Spain; floperdi@itacyl.es; 2 Departamento de Biología Animal, Facultad de Biología, Universidad de Salamanca, 37007 Salamanca, Spain; victordepaz177@usal.es (V.d.P.); lbanos@usal.es (L.B.-P.); 3 Entomology and Botany Unit, Plant Health Laboratory, French Agency for Food, Environmental and Occupational Health & Safety (ANSES), 755 Avenue du Campus Agropolis, CS 30016, FR-34988 Montferrier-sur-Lez Cedex, France; saskia.bastin@anses.fr; 4 Research Group in Invertebrate Diversity and Phylogenetics, Faculty of Biology, Alexandru Ioan Cuza University, Bd. Carol I, Nr. 11, 700506 Iasi, Romania; lucfusu@hotmail.com; 5 Departamento de Ciencias Agroforestales, ETS Ingenierías Agrarias, Universidad de Valladolid, Avenida de Madrid, 57, 34004 Palencia, Spain; mariaisabel.pozo@uva.es

**Keywords:** leek, carrot, parasitoid, *Tamarixia*, *Syrphophagus*, parasitism rates, DNA barcode

## Abstract

Biological control using parasitoids is a key strategy for managing psyllid pests in horticultural crops. This study investigated the parasitoid complex associated with *Bactericera tremblayi* and *Bactericera trigonica,* two economically important psyllid pests of leek and carrot, respectively, in Castile and León (Spain). Nymphs of both psyllid species were collected and reared to assess parasitism; emerging parasitoids were identified morphologically and by DNA barcoding. A total of 969 parasitized nymphs were collected, from which 291 parasitoids emerged, including *Tamarixia pronomus*, *Tamarixia tremblayi*, *Syrphophagus herbidus*, *Syrphophagus* cf. *taeniatus*, *Cheiloneurus* sp. and *Encyrtidae* sp. *Tamarixia pronomus* is reported here for the first time as parasitoids of *B. tremblayi* while *T. tremblayi, S. herbidus,* and *S.* cf. *taeniatus* are newly recorded as parasitoids of *B. trigonica.* Moreover, the two *Syrphophagus* species represent the first records of this genus for Spain. The COI barcode sequences were generated for *T. pronomus*, *T. tremblayi* and *S.* cf. *taeniatus*. The parasitism rate of *S. herbidus* on *B. trigonica* reached a maximum of 23.18%. No significant differences in parasitism by *Tamarixia* species were observed between the two *Bactericera* hosts. Maximum parasitism rates recorded were 32.43% for *T. pronomus* on *B. trigonica*, and approximately 11–12% for *T. tremblayi* on both host species. Total parasitism of *B. tremblayi* in leek crops reached up to 70%, whereas parasitism of *B. trigonica* in carrot remained generally low and poorly synchronized with pest population dynamics. These results highlight a diverse parasitoid complex with substantial natural control of *B. tremblayi* and variable but lower impact on *B. trigonica*, underscoring the relevance of habitat and temporal factors for conservation biological control in these agroecosystems.

## Simple Summary

Parasitoid wasps are important natural enemies that contribute to the biological control of psyllid pests in vegetable crops. This study identified the parasitoid species associated with two major psyllid pests of leek and carrot in Castile and León (Spain). Several new host associations were recorded, including the first record of *Tamarixia pronomus* parasitizing *Bactericera tremblayi*, and the first records of *Tamarixia tremblayi*, *Syrphophagus herbidus*, and *S.* cf. *taeniatus* as parasitoids of *Bactericera trigonica*. In addition, two *Syrphophagus* species are reported for the first time in Spain. DNA barcode sequences were generated for *T. pronomus*, *T. tremblayi*, and *S.* cf. *taeniatus*. Parasitism reached up to 70% in leek crops infested by *B. tremblayi*, whereas it remained lower and less synchronized with *B. trigonica* populations in carrot crops. These findings expand current knowledge of parasitoid diversity and highlight their potential role in the biological control of psyllid pests in vegetable production systems.

## 1. Introduction

Biological control is a pivotal strategy where beneficial organisms are used to manage crop pests by reducing their populations [1]. Parasitoids, as beneficial organisms, play an important role in the complex system of species involved in pest regulation within agricultural ecosystems [2], maintaining a balance between plants and their pests [3]. Parasitoid wasps are the most successful group of insect parasitoids, comprising more than half of the known diversity of Hymenoptera and probably most of the unknown eukaryotic diversity [4,5]. Knowledge of these species is essential for their conservation and provides alternative tools for pest management within an integrated control framework, thereby contributing to sustainable agricultural practices [4,5]. Biological control strategies are commonly categorized as classical, augmentative, or conservation biological control, and can be implemented either separately or in combination [6]. Classical biological control aims at the long-term suppression of invasive pests through the introduction of non-native natural enemies, whereas augmentative biological control involves the mass release of natural enemies to provide temporary crop protection during the production period [6]. Conservation biological control seeks to enhance naturally occurring pest suppression through strategies such as the selection of pesticides that minimize adverse effects on natural enemies and habitat manipulation to increase the abundance and activity of beneficial organisms [6]. These approaches are particularly important because intensive use of chemical insecticides not only promotes the development of insecticide resistance but also disrupts natural enemy communities, reducing biological control efficacy and frequently leading to secondary pest outbreaks [7,8].

Due to the significant agricultural economic impact associated with psyllids as pests or vectors of plant pathogens, considerable attention has been devoted to their control [9]. Classical biological control has been successfully implemented in programs targeting *Diaphorina citri* (Hemiptera: Psylloidea) in California and *Trioza erytreae* (Hemiptera: Psyllidae) in Europe [10,11,12]. Conservation biological control emphasizes the substitution of broad-spectrum chemicals with selective insecticides and the implementation of habitat management to preserve resident beneficial fauna [13,14]. This strategy represents a low-risk alternative that exploits naturally occurring communities by providing essential resources, such as floral food and alternative psyllid prey or hosts within adjacent natural habitats [13,15]. However, the effectiveness of these strategies relies on accurately identifying local parasitoid communities and understanding their phenological synchrony with the target psyllid [1,3]. Therefore, characterizing parasitoid diversity and parasitism rates is essential for developing reliable and sustainable conservation biological control programs [3].

*Bactericera tremblayi* (Wagner) and *Bactericera trigonica* (Hodkinson) (Hemiptera: Triozidae) are two psyllid pests reported in leek (*Allium ampeloprasum* var. *porrum* (L.) J. Gay) and carrot (*Daucus carota* L.), respectively, in Spanish horticultural production areas where they can cause significant economic damage [16,17]. *Bactericera tremblayi*, the onion and leek psyllid, is distributed throughout the Mediterranean region, including Spain [18], where high infestation levels can cause severe foliar and stem damage, leading to reduced crop quality and yield [19]. *Bactericera trigonica* feeds mainly on plants of the family Apiaceae, especially on carrot [20,21,22], and is responsible of the transmission of the phloem-restricted bacterium *Candidatus* Liberibacter solanacearum in this crop in Spain [23], which is associated with severe vegetative disorders in affected plants [24,25]. The population density of these two species tends to increase during the summer months, but maximum population peaks are observed in late-season crop cycles, spanning from September to November, driven by the seasonal biology of the psyllids and the phenology of late-season host crops [16,17].

Research on parasitoids associated with pest species of the genus *Bactericera* is still scarce. For *B. tremblayi*, *Tamarixia tremblayi* (Domenichini) has been reported as a parasitoid on *Allium cepa* L. [26,27], whereas *Tamarixia monesus* (Walker) has been identified on *Brassica oleracea* var. *capitata* L. in Iran [28]. Regarding *B. trigonica*, only *T. pronomus* (Walker) has been identified as a parasitoid in Tunisia [29]. Nevertheless, the diversity and distribution of parasitoids associated with *Bactericera* species remain poorly studied, largely due to the inherent difficulties involved in accurately identifying parasitoid species. In this context, molecular approaches, particularly DNA barcoding, represent a valuable complementary tool for parasitoid identification, especially for hymenopteran species that are difficult to identify reliably based solely on morphology because of their small size, high morphological similarity, and the scarcity of diagnostic characters [3]. DNA barcoding, based on a short standardized DNA sequence, can therefore improve taxonomic resolution and validate morphological identifications [30,31,32,33,34]. Cytochrome c oxidase I gene (COI) has been proposed as a universal barcode for all animals, with broad consensus that COI will successfully identify the majority of animal species “http://www.barcodinglife.com” (accessed on 2 June 2025) [30,35,36]. Nevertheless, the limited availability of reference sequences for many parasitoid taxa in public databases still constrains accurate species identification and highlights the need to expand barcode reference libraries [3].

The main objective of this work was to improve the knowledge of the natural enemies associated with these two psyllid species. The specific aims of this work were to inventory the parasitoid species associated with *B. tremblayi* and *B. trigonica* in leek and carrot fields within the horticultural region of Castile and Leon, integrating morphological identification with DNA barcoding; to provide COI barcode sequences of the parasitoid species; and to estimate parasitism rates by parasitoid species and total parasitism rate for two host–parasitoid complexes studied, *B. tremblayi* in leek and *B. trigonica* in carrot.

## 2. Materials and Methods

### 2.1. Study Area and Sampling Methodology

**Figure 1:**
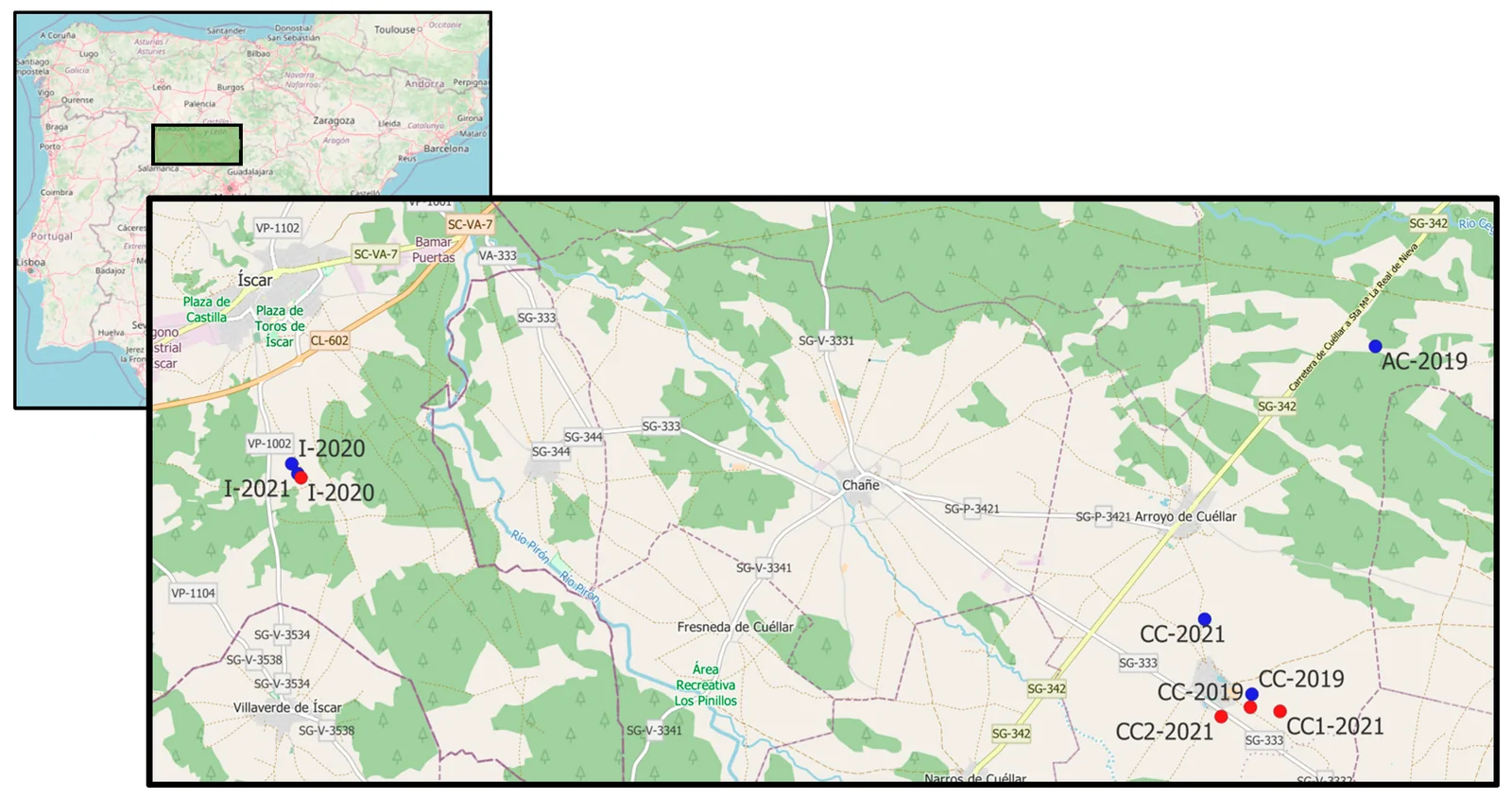
Locations of leek fields, associated with *B. tremblayi* (red), and carrot fields, associated with *B. trigonica* (blue), within the study area in Castile and León from 2019 to 2021. (Abbreviations are as presented in Table 1).

The survey was conducted in the Castile and Leon region, which accounts for nearly half of Spain’s leek and carrot production [37,38]. Specifically, the study area is located near the border between the provinces of Valladolid and Segovia, the region’s primary horticultural growing area (Figure 1). The landscape is dominated by agricultural land, interspersed with semi-natural patches of pine forest comprising two species: *Pinus pinaster* Aiton and *Pinus pinea* L. Leek and carrot study plots ranged in size from one to five hectares and were managed using organic farming practices. A total of four and five late season plots were monitored in leek and carrot crops, respectively, during the years 2019, 2020, and 2021 (Table S1). Plots were visited once and up to three times from September until the end of the growing season when the presence of immature forms of *B. tremblayi* and *B. trigonica* in leek and carrot fields is highest [16,17]. The number of visits varied depending on the timing of the crop harvest, which ranged from September (early season) to December (late season).

Additionally, associated with *B. trigonica,* a single male specimen of *Cheiloneurus* was identified but could not be classified beyond the genus level, remaining designated as *Cheiloneurus sp.* Four encyrtids, belonging to the genus *Syrphophagus*, were tentatively identified as *Syrphophagus* cf. *taeniatus* (Förster, 1861). Finally, one male encyrtid specimen that could not be confidently assigned to any genus was recorded as *Encyrtidae* sp.

Each sample, categorized by plot and date, consisted of five sets of 20 leaves collected from 100 randomly selected plants. Sampling was conducted across the entire plot, excluding the area within 12 m of the borders. The samples were then placed in plastic zip-lock bags and stored at 4 °C in the laboratory until inspection, which was carried out within the days immediately following collection.

Leaves were examined with a Nikon SMZ1000 Zoom stereo microscope (Nikon Corporation, Tokyo, Japan) and the number of psyllid eggs and nymphs per leaf was recorded. Nymphs were categorized by developmental stage, ranging from first to fifth instar. Nymphs from the third to fifth instar (N3–N5), were isolated in 1.5 mL drilled Eppendorf tubes containing a leaf fragment and a drop of mead. These tubes were monitored every 3–4 days until either an adult parasitoid emerged or the psyllid nymph died. When parasitoid development occurred, emerged individuals were collected and preserved in 70% ethanol for subsequent sorting and identification. Dead psyllid nymphs were examined under a stereo microscope to confirm the presence or absence of parasitoid mummies.

### 2.2. Morphological Identification

Prior to species identification, parasitoid specimens were sorted into families, following Goulet and Hubber [39], and then into morphospecies. After the completion of the DNA extraction process the specimens were mounted. Several specimens from each morphospecies were dissected and slide-mounted in Canada balsam using the method described by Noyes [40]. The remaining individuals were dried using hexamethyldisilazane (HMDS) according to the procedure outlined by Heraty and Hawks [41] and then mounted on cardboard points.

Only parasitoids from the families Eulophidae and Encyrtidae (*Hymenoptera*: *Chalcidoidea*) emerged. Eulophids were sorted into genera and identified to species using Graham [42]. Encyrtids were sorted into genera following Noyes [43] and Trjapitzin [44]. One specimen could not be assigned to any genus with certainty, and a male *Cheiloneurus* (Hymenoptera, Encyrtidae) could not be identified further, remaining as *Encyrtidae* sp. and *Cheiloneurus* sp., respectively. All other encyrtids belonging to the genus *Syrphophagus* (Hymenoptera: Encyrtidae) were identified to species based on early reports [44,45,46,47]. All slide-mounted and point-mounted specimens were identified to account for possible errors during morphospecies sorting. Voucher specimens have been deposited at the Instituto Tecnológico Agrario de Castilla y León (Spain) and AICF (Lucian Fusu Collection, Al. I. Cuza University of Iaşi, Romania, Table S2).

### 2.3. DNA Barcoding

DNA was extracted from three to six specimens per morphologically identified species using a non-destructive Chelex protocol, following the method described by Casquet et al. [48]. *Cheiloneurus* sp., with a single individual, was excluded from DNA analysis and only used for morphological identification. The standard DNA barcode region at the 5′ end of the mitochondrial cytochrome c oxidase I gene (COI-5P) was amplified using the universal primer pair, LCO-1490 and HCOI-2198 [49]. PCR amplification was performed in a 40 μL final reaction including 1 μL of each primer, 14 μL of Sigma dH_2_O, 20 μL REDExtract-N-Amp PCR ReadyMix (Sigma Aldrich, St. Louis, MO, USA), and 4 μL of genomic DNA. Polymerase chain reactions (PCRs) were carried out in an Applied Biosystems 7500 Fast Real-Time PCR System applying the following thermal step: initial denaturation for 4 min at 94 °C, followed by 39 cycles of 30 s at 94 °C, 30 s at annealing temperature of 48 °C, and 45 s at 72 °C, then a final extension step of 10 min at 72 °C. PCR products were enzymatically cleaned adding 0.83 U rAPid alkaline phosphatase (EURx, Gdansk, Poland) and 0.1 U exonuclease I (EURx, Gdansk, Poland) per 5 μL of PCR products for 30 min at 37 °C followed by 5 min at 95 °C. The sequencing reaction was carried out utilizing the ABI 3730 DNA Analyzer (Applied Biosystems, Foster City, CA, USA).

Forward and reverse sequences were inspected, edited, assembled into consensus sequences, and subsequently aligned using MUSCLE [50] in MEGA version 12.1 [51,52]. Consensus sequences were compared using BLAST 2.15.0 through the NCBI web interface (accessed on 15 January 2024) and the Identification Engine Tool (IDS) “https://boldsystems.org/index.php/IDS_OpenIdEngine” (accessed on 15 January 2024) against the known sequences in the Genbank database “http://blast.ncbi.nlm.nih.gov/Blast.cgi” (accessed on 15 January 2024) and the Barcode of Life Data System (BOLD Systems), respectively. As the barcode regions of all the species detected in the morphological identification are not available in any database, the sequences of COI fragments were used for phylogenetic analyses. The sequences were aligned, by codon, using MUSCLE [50] implemented in MEGA v.12.1 [51]. Neighbor-Joining algorithms were then conducted using a Kimura-2-parameter (K2P) substitution model with 1000 bootstrap replications in MEGA v.12.1. The sequences obtained in this study were deposited in the Genbank database under the accession numbers PV132258 (*Tamarixia pronomus*), PV132259 (*Tamarixia tremblayi*), PV132260 and PV132261 (*Syrphophagus* cf. *taeniatus*).

### 2.4. Parasitism Rates

Parasitism rates were calculated by plot and date. The parasitism exhibited by species (Ps) was calculated as Ps = [(number of N3–N5 psyllid nymphs parasitized by each parasitoid species/total number of N3–N5 psyllid nymphs) × 100]. The total parasitism rate (Pt) was calculated as Pt = [(number of parasitized N3–N5 psyllid nymphs/total number of N3–N5 psyllid nymphs) × 100]. Parasitized psyllid nymphs were defined as those from which either parasitoid or hyperparasitoid adults emerged, as well as those in which parasitoids were present but did not emerge, evidenced by the observation of mummified nymphs [53]. Finally, effective parasitism rate (Pe) were calculated as Pe = [(number of adult emerged parasitoids/total number of N3–N5 psyllid nymphs) × 100]. The calculation of effective parasitism considered both adult parasitoids that emerged in the laboratory and nymphs with emergence holes, indicative of prior adult emergence in the field.

### 2.5. Data Analysis

Generalized linear mixed models (GLMMs) with a binomial error distribution and logit link were used to analyze parasitism rates. Separate models were fitted for each parasitism measure. In each model, the response variable was specified as a matrix of successes and failures, where successes represented the number of parasitized nymphs according to the parasitism measure analyzed, and failures represented the corresponding number of non-parasitized nymphs within each sampling unit. For total (Pt) and effective parasitism (Pe), as well as the parasitism of the species *S. herbidus* which was recorded exclusively from *B. trigonica*, separate intercept-only models were fitted for each host species. For parasitism by the generalist parasitoids *T. tremblayi* and *T. pronomus*, which parasitize both *B. tremblayi* and *B. trigonica*, host species was included as a fixed effect to evaluate differences between hosts. In all models, Year, Plot nested within Year, and Date nested within Plot and Year were included as random effects to account for the hierarchical sampling design.

Differences in Pt, Pe, and the number of N3–N5 psyllid nymphs per leaf among sampling dates within each plot and year were evaluated using a non-parametric analysis of variance, specifically the Kruskal–Wallis test, with a significance level of *p* < 0.05.

All statistical analyses were conducted using R software, version 4.6.1. (R Development Core Team, 2026).

## 3. Results

### 3.1. Parasitoid Inventory

Over the course of the three-year study period, a total of 969 parasitized nymphs were collected from *B. tremblayi* (634) and *B. trigonica* (335). Among these, a total of 291 parasitoids emerged, comprising 136 from *B. tremblayi* and 155 from *B. trigonica* nymphs. The remainder of the 678 parasitoids did not develop into adult parasitoids (Table 1).

*Syrphophagus herbidus* (Dalman) (Hymenoptera: Encyrtidae) was identified primarily in association with *B. trigonica* nymphs, with 76 out of 77 individuals emerging from this host, confirming it as the predominant parasitoid species in carrot crops. This species accounted for 49.03% of all parasitoids observed on this host and exhibited a notable emergence peak during 2019 (Table 1).

*Tamarixia pronomus* was present with 85 and 45 individuals in *B. tremblayi* and *B. trigonica*, accounting for 62.50% of the collected parasitoids; meanwhile in *B. trigonica* psyllid it comprised 29.03% of the total identified individuals respectively (Table 1). The species was detected starting from September, but its prevalence notably increased from October onwards (Table 1). Similarly, *T. tremblayi* was observed in 45 and 27 individuals of *B. tremblayi* and *B. trigonica*, accounting for 33.09% and 17.42% of the total individuals identified respectively. An additional six specimens could only be classified at the genus level as *Tamarixia* sp.

**Figure 2:**
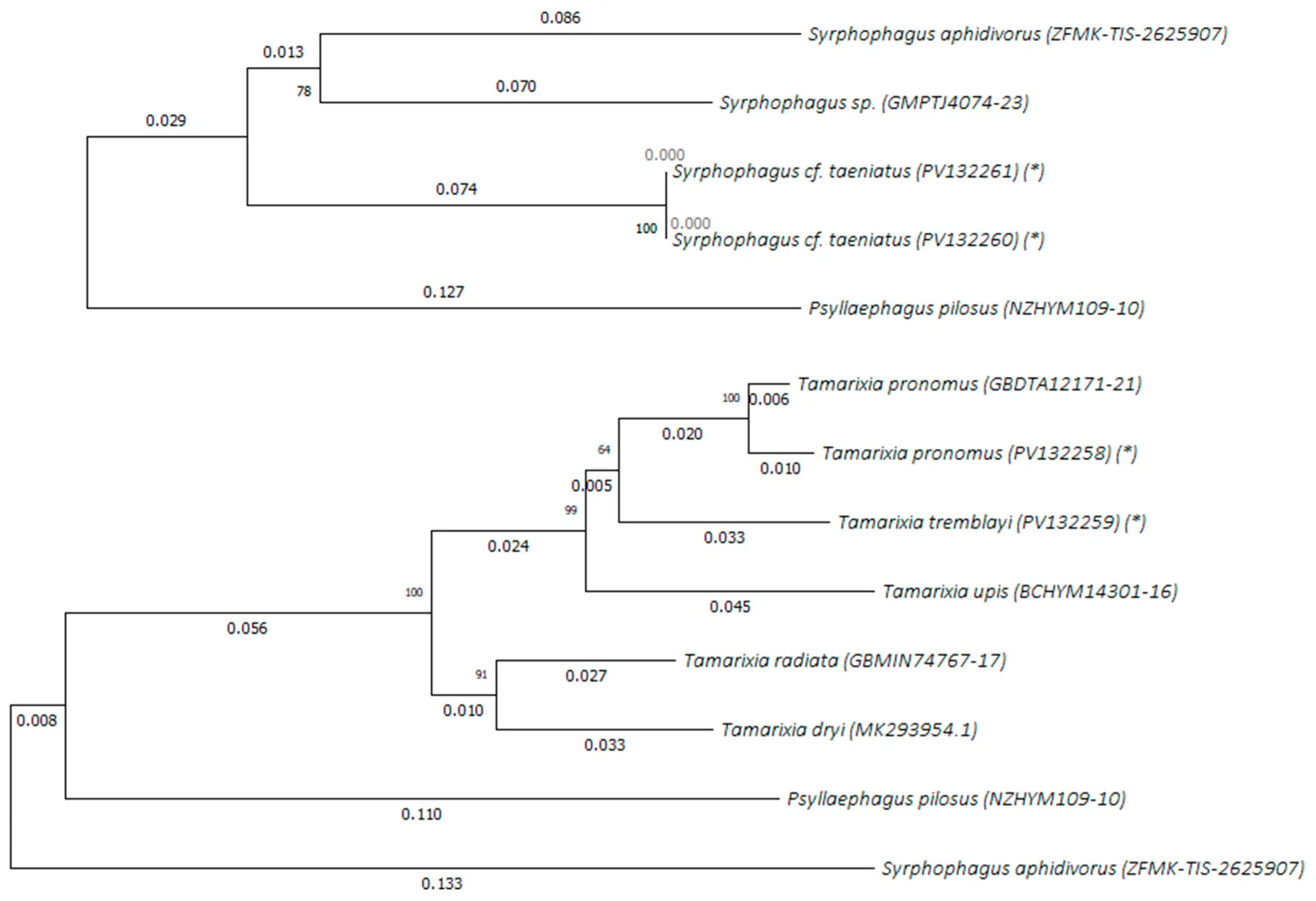
Neighbor-Joining tree for *Syrphophagus* cf. *taeniatus* and *Tamarixia* spp. based on COI sequences, using Kimura-2-parameter distance. Bootstrap support values are based on 1000 replications. Numbers on branches are branch lengths. GenBank accession numbers for the retrieved sequences are provided in parentheses. Sequences generated from this study are denoted with an asterisk (*).

DNA barcode sequences obtained for *T. pronomus*, *T. tremblayi*, and *S.* cf. *taeniatus* confirmed the morphological identification of these species and provide new molecular reference sequences for taxa that remain poorly represented in public databases. The amplified COI barcode sequences were 630 bp in length. In the case of *S. herbidus*, the obtained sequences displayed signal inconsistencies, preventing a clear and reliable interpretation of the data. For *T. pronomus*, the BOLD Systems database currently includes 49 entries from Egypt and Germany [54], although no sequences from Spain have been deposited to date. The sequence obtained in this study exhibited 99% similarity to the sequence of *T. pronomus* (Germany, Bavaria) deposited in the Bold systems databases (Figure 2). The sequence of *T. tremblayi* shared 93.56% identity with the two sequences or *T. pronomus* used in the phylogenetic analysis and 91.59 and 89.93% identity with *Tamarixia upis* (Walker) and *T. dryi* sequences, respectively (Table S3). The Neighbor-Joining tree of the *Tamarixia* species showed two clusters (Figure 2). The first cluster grouped together *T. tremblayi, T. pronomus* and *T. upis* while the second cluster is formed by *T. dryi* and *T. radiata*. The sequences of *S.* cf. *taeniatus* displayed 82.6 and 84.42% similarity when compared to *Syrphophagus aphidivorus* and *Syrphophagus* sp., respectively, as retrieved from the Bold Systems databases (Figure 2, Table S3). The Neighbor-Joining tree constructed for the *Syrphophagus* species revealed two distinct clusters (Figure 2). One cluster comprised *Syrphophagus* sp. and *S. aphidivorus*, while the second cluster contained the two *S.* cf. *taeniatus* specimens identified in this study.

### 3.2. Parasitism Associated with Parasitic Species

**Figure 3:**
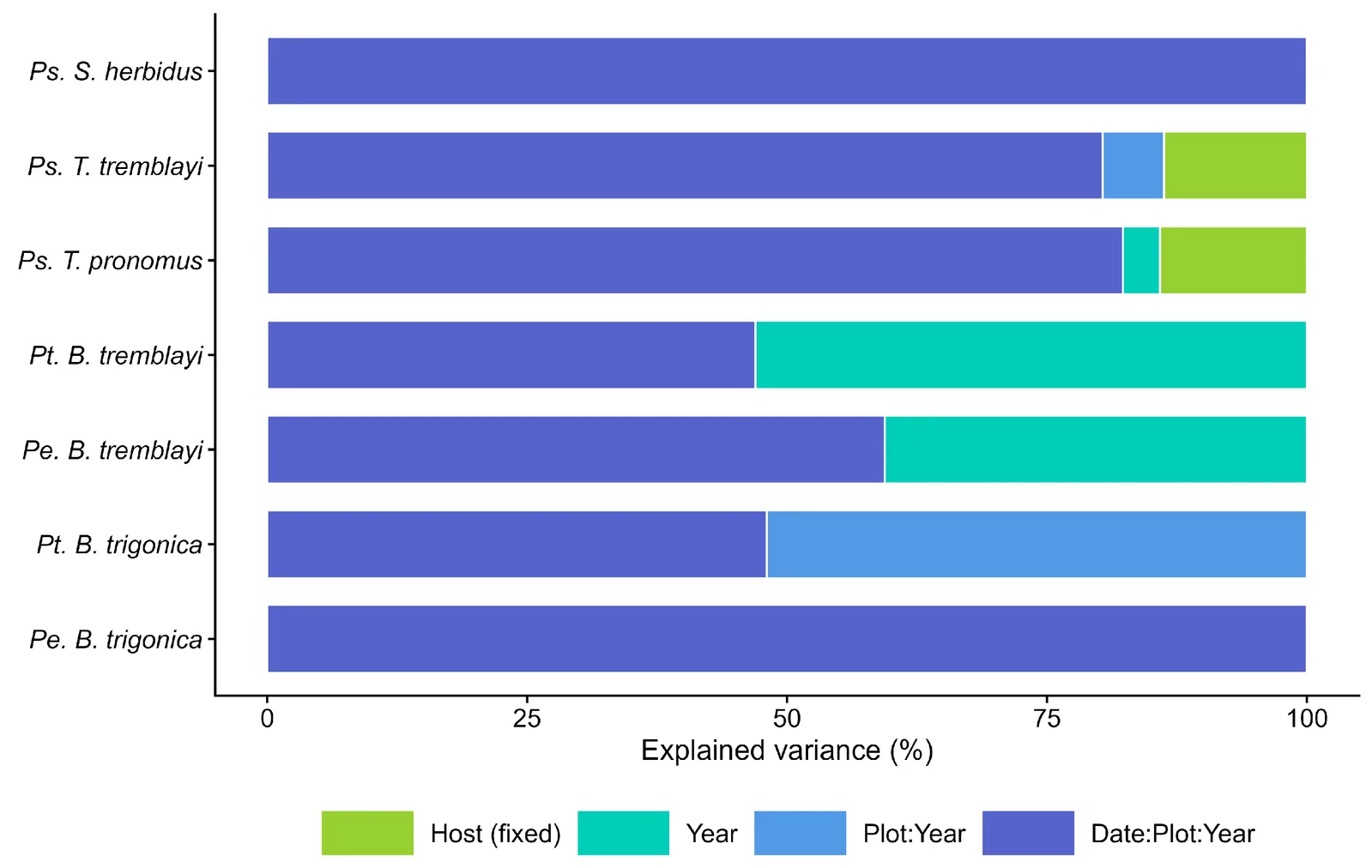
Partitioning of variance in parasitism rates explained by fixed (host species) and random effects (Date:Plot:Year, Plot:Year and Year). Variables shown include parasitism attributed to each parasitoid species (Ps), total parasitism (Pt) and effective parasitism rate (Pe), both recorded per host species (*B. trigonica* and *B. tremblayi*).

Parasitism by *S. herbidus* was predominantly explained by temporal differences between sampling dates (Figure 3). Specifically, peak occurrences of this parasitoid species were notably concentrated in mid-September in 2019 with the highest parasitism rate reaching 23.18% at Arroyo de Cuéllar (Table 1). Subsequent emergences of this species were recorded in mid-October, although with a lower Ps.

Data analysis of *T. pronomus* and *T. tremblayi* revealed that hosts did not exert a statistically significant effect on parasitism rates (*T. pronomus*: χ^2^ = 2.14, *p* = 0.143; *T. tremblayi*: χ^2^ = 1.76, *p* = 0.185). In both species, the fixed effect alone explained only a low proportion of the variance (R^2^m = 0.14 and 0.13, respectively), whereas the full models including random effects accounted for nearly all of the explained variance (R^2^c = 0.98 in both cases), indicating that most variation was attributable to the random-effects structure. Variance partitioning showed that temporal variation associated with sampling date nested within plot and year was the main source of random variation, followed by plot nested within year, while the contribution of year alone was negligible (Figure 3). The maximum parasitism rate observed for *T. pronomus* was 32.43% in carrot at the Iscar plot in October 2021, whereas maximum parasitism rates for *T. tremblayi* were approximately 11–12% for both host species (Table 1).

Parasitism exhibited by the rest of the species identified has not been calculated due to the very low number of individuals, with a negligible Ps.

### 3.3. Parasitism Rates of Bactericera tremblayi

Variance partitioning among random effects indicated that most of the variability of total parasitism (Pt) was associated with Year and Date:Plot:Year component, whereas Plot:Year variation did not contribute to the explained variance (Figure 3). The trend in effective parasitism (Pe) was similar to that of Pt, indicating that most of the variability of Pe was associated with Date:Plot:Year and Year (Figure 3).

**Figure 4:**
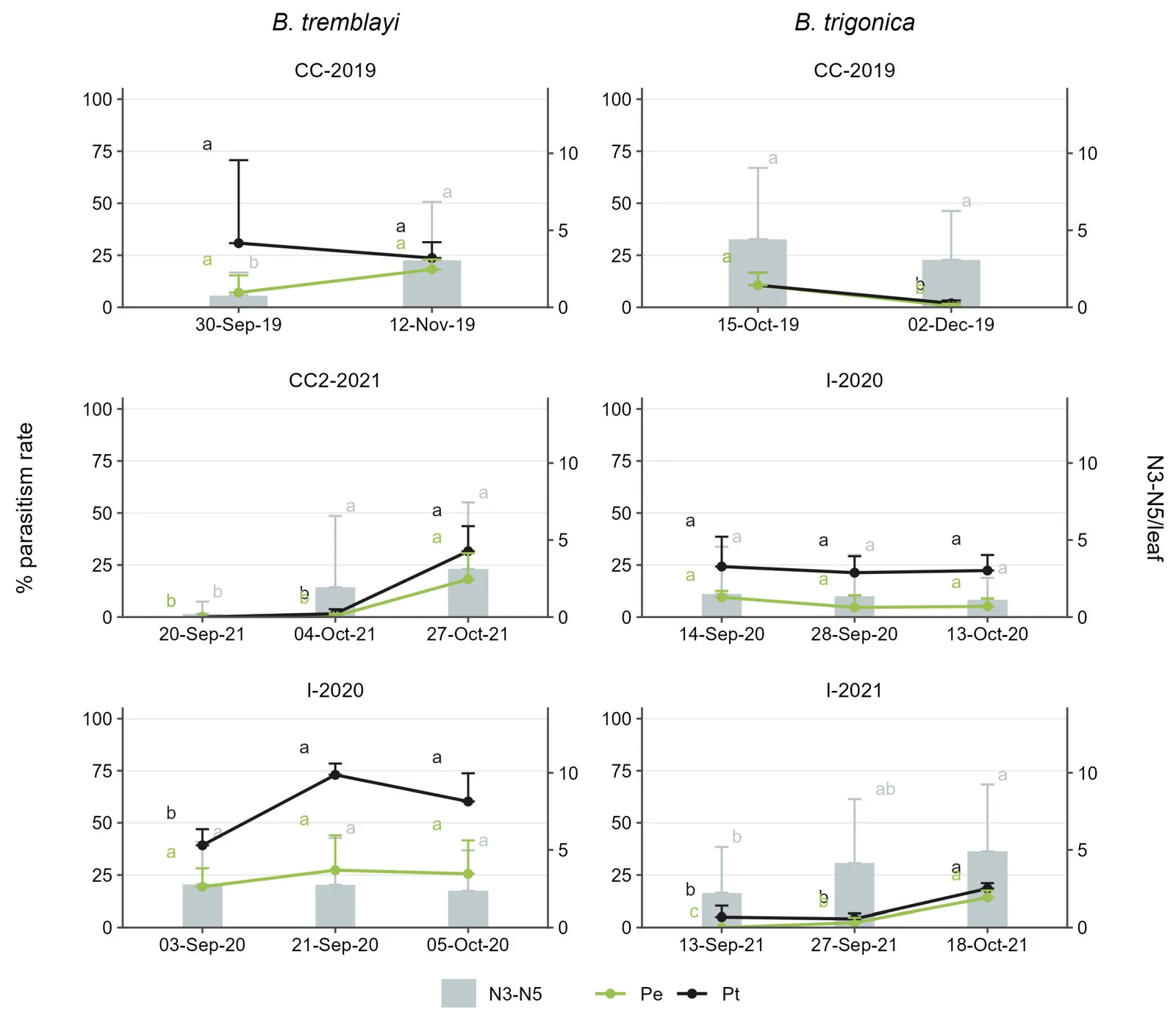
Parasitism rates (first axis), Pt (Total parasitism) and Pe (Effective parasitism), and number of N3–N5 nymphs per leaf (second axis) categorized by host species, plot and date. Different letters show significant differences by each variable between dates (Kruskal–Wallis test, *p* < 0.05).

Marked interannual variation was observed in parasitism levels, for both Pt and Pe, coinciding with fluctuations in the pest population dynamics (Table 2). The lowest parasitism values were recorded in 2021 (Table 2). Temporally, significant differences by dates were detected primarily in Pt during 2020 and 2021, particularly between early and later sampling dates in both years (Figure 4). These differences paralleled pest levels, suggesting synchronization between pest abundance and parasitism rates.

### 3.4. Parasitism rates of Bactericera trigonica

Variability in Pt in *B. trigonica* was attributable to Date and Plot, suggesting that the observed variability is driven by temporal and spatial levels (Figure 3). However, variance components indicated that all the explained variability in Pe in carrot was associated with Date, whereas both Plot and Year showed negligible contributions to the total variance (Figure 3).

Parasitism levels for Pt and Pe were comparable overall but remained low, with marked differences between years, whereas pest levels were high, suggesting low synchrony with the pest population (Table 2) in contrast to *B. tremblayi*. Parasitism values were higher in 2020 and remained stable across sampling dates (Figure 4). Regarding temporal variation, higher Pt and Pe values were observed during the month of October, showing significant differences compared to later sampling dates in December 2019 and to earlier dates in 2021.

## 4. Discussion

Biological control of psyllids by parasitism is crucial for sustainable effective management of pest populations. Although classical biological control has proven to be a highly effective strategy for managing economically important psyllid pests through the introduction and mass release of host-specific parasitoids of the genus *Tamarixia* [55,56,57], conservation biological control is increasingly recognized as a sustainable approach for enhancing naturally occurring parasitoids [58]. Nevertheless, the diversity and distribution of parasitoids associated with *Bactericera* species are still poorly understood. Accurate identification of the parasitoid species present in the agroecosystem [59] and determining host-specific parasitism levels are crucial for understanding their ecological roles and optimizing their application in integrated pest management strategies [60].

Based on the inventory results obtained in the present study, two species of the genus *Tamarixia* were identified. This genus is important in classic biological control, due to a high degree of host specificity [61]. Most species act as ectoparasitoids, but in some cases endoparasitism has been reported [62]. Several species of *Tamarixia* are considered in biological control programs of certain psyllids, such as *Bactericera cockerelli* (Šulc) [12,63], *D. citri* [11,64], and *T. erytreae* [10,57,65,66].

*Tamarixia pronomus* was identified for the first time as a parasitoid of *B. tremblayi*. Regarding *B. trigonica*, its status as a parasitoid host was confirmed, which is consistent with previous records from Tunisia [29]. Notably, this research represents the first documented occurrence of *T. pronomus* on mainland Spain. Conversely, the presence of *T. tremblayi* on mainland Spain had already been confirmed as a parasitoid of *B. tremblayi* [26]. However, this parasitoid species was recorded for the first time parasitizing *B. trigonica*. Lastly, *T. monesus*, which has been reported in other studies as parasitizing *B. tremblayi* [28], was not observed in the present study.

The genus *Syrphophagus* was the second most abundant group among the parasitoid species. Two species were identified: *S. herbidus* and *S.* cf. *taeniatus.* To date, no presence of these two species has been reported in Spain [62]. Consequently, this study provides the first record of *S. herbidus* parasitizing *B. trigonica* and the first documented occurrence of this species in the country. Regarding *S.* cf. *taeniatus*, should its identification be confirmed, this finding would also be the first national record and the first reported case of this species parasitizing *B. trigonica*. The specimens from Spain agree very well with other identified specimens from this species, except the sensory area of the clava which is shorter (J. Noyes, personal communication). Additionally, this study presents the first DNA barcode sequence data for *S.* cf. *taeniatus*. Most species of the genus *Syrphophagus* have been reported as hyperparasitoids of Diptera, aphids, and jumping plant lice [67,68,69], including species of *Tamarixia* [70,71,72,73], although some species can also act as primary parasitoids of diaspidids and psyllids [62,74]. In this study, both *S. herbidus* and *S.* cf. *taeniatus* were found exclusively associated with *B. trigonica*, which could be consistent with a role as primary parasitoids in this system. Under this hypothesis, the single record of *S. herbidus* from *B. tremblayi* could be explained by the occasional presence of *B. trigonica* nymphs on leek [17], given that species of the genus *Bactericera* exhibit morphological similarities and exploit a wide range of herbaceous host plants [19]. However, this hypothesis is not conclusive. Likewise, the relatively low emergence of *Tamarixia* spp., particularly during the period when *S. herbidus* was detected, could also be consistent with hyperparasitism, in which case the single record from *B. tremblayi* could simply reflect occasional hyperparasitism by *S. herbidus* parasiting *Tamarixia* in this host, whereas host preferences or differences in host suitability [75] could explain its predominant association with *B. trigonica* despite the presence of *Tamarixia* parasitoids in both psyllid species Therefore, the trophic role of *S. herbidus* and *S.* cf. *taeniatus* in this agroecosystem remains uncertain, and further studies are needed to determine whether these species act as primary parasitoids, hyperparasitoids, or both.

Finally, a single specimen of *Cheiloneurus* was identified in this study. To date, four species of this genus have been documented in Spain: *Cheiloneurus boldyrevi* Trjapitzin and Agekian, *Cheiloneurus claviger* Thomson, *Cheiloneurus elegans* (Dalman) and *Cheiloneurus paralia* (Walker) [62]. All known species of *Cheiloneurus* with documented biology have proven to be hyperparasitoids, and some have been reported as secondary of psyllid parasitoids associated with Psylloidea, including those attacking *D. citri* and *T. erytreae* [76,77,78,79,80]. Most described hyperparasitoids are solitary, perhaps due to their phylogenetic closeness and similar size to their hosts [81]. By attacking primary parasitoids, hyperparasitoids may affect herbivore population dynamics, and they have been identified as a major challenge in biological control [81]. Conversely, other researchers suggest that hyperparasitoids can facilitate biological control by stabilizing host–parasitoid and parasitoid–parasitoid dynamics [67]. From a biological control perspective, hyperparasitoids play a crucial role in mediating interactions between primary parasitoids [67]. In our study, with the presence of only one *Cheiloneurus* species as a potential hyperparasitoid, the hyperparasitism rate was low, with no significant impact on pest control.

Parasitism rates serve as a key indicator of the potential efficacy of parasitoid species as biological control agents, and their magnitude is known to exhibit substantial variation across different host–parasitoid systems. In the present study, the parasitism rates recorded for both *Tamarixia* species differed between the two psyllid host species examined. Parasitism rates varied between the two parasitoid species, with *T. pronomus* reaching higher maximum values than *T. tremblayi*. Notably, the parasitism rate of *T. pronomus* appeared to be higher than that of *T. tremblayi*. However, these rates were generally lower than those reported by Othmen et al. [29] for *T. pronomus* parasitizing *B. trigonica* under field conditions. These authors reported marked variability depending on the developmental stage of the host nymphs, with maximum parasitism rates of 23.53% and 56.86% in second and third instar nymphs, respectively, and values exceeding 80% in fourth and fifth instar nymphs. This interspecific variability is consistent with the broad range of parasitism rates reported within the genus *Tamarixia*, which have been shown to vary from 20% to 80% [10,82,83,84]. In particular, certain species within the genus *Tamarixia*, such as *T. radiata* and *T. dryi*, due to their high parasitism rates on their respective host species, have been widely used as classical biological control agents against pest psyllid species, demonstrating notable efficacy [55,56,57].

The occurrence of *S. herbidus* was highly specific and temporally restricted, being observed almost exclusively in 2019. During that year, it emerged as the dominant parasitoid species associated with *B. trigonica*, reaching a parasitism rate of 23.18%. In contrast, *T. pronomus* and *T. tremblayi* exhibited substantially lower parasitism rates of 0.83% and 4.72%, respectively. The other congeneric species, *S.* cf. *taeniatus*, appeared synchronously in the same plot and during the same period, although at a considerably lower abundance, with only four individuals recorded. Regarding the parasitoid community recovered from *B. trigonica*, *S. herbidus* accounted for nearly 78% of all parasitoid individuals collected from this host during that year. However, to date, no data are available concerning the abundance of *S. herbidus* in other parasitism studies. With respect to *S.* cf. *taeniatus*, previous studies have reported its relative abundance among secondary parasitoids, with values ranging from 7% to 67.61% [67,68,69,85]. These findings highlight the need for further research to elucidate the ecological factors driving such marked geographical and temporal disparities.

Although parasitism rates by species constitute a critical metric in classical biological control, within the framework of conservation biological control, the total parasitism rate, which integrates all species, holds greater significance. In the present study, particularly in leek plots during 2020, parasitism of *B. tremblayi* reached maximum values of up to 70%. Comparable levels of field parasitism have been reported for other psyllid species. For instance, parasitism rates in *Agonoscena pistaciae* Burckhardt and Lauterer (Hemiptera: Sternorrhyncha: Psyllidae) showed considerable variability under field conditions, although maximum values were similar to those observed in our study [86]. Similarly, for *T. erytreae*, field studies have reported parasitism rates ranging between 40% and 70% [10].

As well as parasitism rates associated with the studied parasitoid species, total parasitism in both hosts was highly variable, primarily driven by temporal factors. These temporal patterns are driven by environmental variables operating under field conditions, with temperature being one of the most important abiotic factors influencing the survival, development, and reproduction of both hosts and parasitoids [87], as well as the degree of synchrony between their respective life cycles. Key aspects of this synchrony include whether host generations are discrete or overlapping, the timing of parasitoid activity relative to the host’s vulnerable period, and the development time of the parasitoid compared to that of the host [3]. Taxonomic affinity have also been demonstrated to be an important host selection factor for parasitoid species [88]. In this case, the two *Tamarixia* species identified were observed targeting both host species of *Bactericera.* Parasitism rates varied across the two pests, but host species identity did not appear to influence parasitism rates in either *Tamarixia* species. Although species of the genus *Tamarixia* have a high degree host specificity [61], both species belong to the *Bactericera nigricornis* Förster complex and exhibit very similar morphological traits [19,20]. In the case of *T. pronomus*, it has been identified in several psyllid hosts including *Bactericera kratochvili* Vondráček, *Trioza apicalis* Foerster, *Trioza centranthi* (Vallot), *Trioza urticae* [42,89] and the jumping plant-louse, *Bactericera crithmi* (Löw) [90]. Nevertheless, the number of host species reported for *T. tremblayi* to date is smaller, with records limited to *B. tremblayi*. The coexistence of these two *Tamarixia* species within the same habitat, coupled with their shared utilization of two host species, represents a viable opportunity for their integration into a biological control program.

Although the two *Syrphophagus* species in our study were found exclusively associated with *B. trigonica*, most species of this genus are known to be primary or secondary parasitoids of pupae from the diverse insect orders [44,62,68,69,74,86]. This suggests that for these species, host specificity is low, and other potential hosts may be available for parasitism. Notably, generalist parasitoid species are key to conservation biological control, as they can persist in the ecosystem even when the pest is scarce, filling niches left unoccupied by specialists and contributing functional complementarity to the complex [3].

The two host–parasitoid complexes studied exhibited contrasting synchrony patterns. In *B. tremblayi*, parasitism rates were synchronized with pest population levels, whereas this pattern was not observed in *B. trigonica*. Such differences highlight the complexity of factors governing parasitoid dynamics, which extend beyond host abundance to include interactions among natural enemies and habitat-related characteristics of the agroecosystem [3]. Indeed, the spatial variation detected in parasitism rates of *B. trigonica* points to the importance of local and landscape-scale conditions. Factors such as local crop management practices, agricultural intensification at landscape and regional scale, and landscape composition are known to influence parasitoid populations and their effectiveness [91,92]. Many of these practices reduce uncultivated habitats, which are essential for natural enemies by providing alternative hosts or prey, refuges, overwintering sites, and floral resources [93], as well as carbohydrates for hymenopteran parasitoids [94,95]. Although psyllid honeydew provides an abundant carbohydrate source for parasitoids and predators during the cropping season [66,96,97], agricultural intensification eliminates adventitious flora and annual plants, thereby removing alternative resources, refuges, and overwintering sites.

Investigating the factors behind the high levels of parasitism observed in the study plots can provide valuable insights for the development of conservation biological control strategies. Such strategies would enhance the sustainability of agricultural systems by minimizing practices that adversely affect the biological communities within agroecosystems. Additionally, within the context of conservation biological control, the parasitism processes involve the participation of multiple species. While this complexity may initially pose challenges in analyzing how each factor differentially impacts these species, it also represents a strength. Agroecosystems with higher biodiversity inherently offer greater opportunities for ecosystem functionality and increased resilience, thereby supporting more stable and sustainable agricultural practices.

## 5. Conclusions

This study confirms the occurrence of parasitism by multiple hymenopteran species associated with *Bactericera* spp. in horticultural crops in Spain, highlighting their potential role in conservation biological control programs. Parasitism rates showed marked temporal and spatial variability, with generally higher levels observed in leek fields compared to carrot plots, indicating a strong influence of numerous factors such as the species involved, environmental conditions, crop phenology, and local management practices. These patterns emphasize the importance of incorporating temporal dynamics and landscape structure when designing strategies to enhance parasitoid activity in agroecosystems. Further research is needed to clarify the ecological role of each parasitoid species and to identify habitat features that support high and stable parasitism levels. Such knowledge would improve the reliability and effectiveness of the biological control of psyllid pests in Mediterranean horticultural systems.

## Figures and Tables

**Table 1:** Number of examined and parasitized nymphs and emerged parasitoids of *B. tremblayi* and *B. trigonica*, and parasitism rates by parasitoid species (Ps) by date and plot. Total numbers and percentages of parasitoid species by host. Castile and León, Spain, 2019 to 2021.

					*S. herbidus*		*T. pronomus*		*T. tremblayi*	
Plot/Locality	Date	*N* *nymphs examined*	*N* *parasitized nymphs*	*N* *parasitoid species*	*N* *emerged*	*Ps (%)*	*N* *emerged*	*Ps (%)*	*N* *emerged*	*Ps (%)*
*Bactericera tremblayi*										
CC-2019/Campo de Cuéllar	30-Sep-19	41	8	4	0	0.00	2	4.88	2	4.88
	12-Nov-19	282	61	44	0	0.00	35	12.41	6	2.13
I-2020/Iscar	03-Sep-20	277	112	44	1	0.36	9	3.25	32	11.55
	21-Sep-20	274	203	2	0	0.00	0	0.00	2	0.73
	05-Oct-20	235	142	2	0	0.00	0	0.00	2	0.85
CC1-2021/Campo de Cuéllar	22-Sep-21	32	1	0	0	0.00	0	0.00	0	0.00
CC2-2021/Campo de Cuéllar	20-Sep-21	22	0	0	0	0.00	0	0.00	0	0.00
	04-Oct-21	203	4	1	0	0.00	1	0.49	0	0.00
	27-Oct-21	328	103	39	0	0.00	38	11.59	1	0.30
	**Total sum**	***1694***	***634***	***136***	***1***		***85***		***45***	
**Percentage of parasitoid species by host**					***0.74***		***62.50***		***33.09***	
*Bactericera trigonica*										
AC-2019/Arroyo de Cuéllar	16-Sep-19	233	91	71	54	23.18	0	0.00	11	4.72
CC-2019/Campo de Cuéllar	15-Oct-19	242	22	22	20	8.26	2	0.83	0	0.00
	02-Dec-19	264	5	2	0	0.00	2	0.76	0	0.00
I-2020/Iscar	14-Sep-20	151	36	2	1	0.66	0	0.00	1	0.66
	28-Sep-20	136	32	0	0	0.00	0	0.00	0	0.00
	13-Oct-20	111	23	0	0	0.00	0	0.00	0	0.00
CC-2021/Campo de Cuéllar	06-Sep-21	99	3	0	0	0.00	0	0.00	0	0.00
I-2021/Iscar	13-Sep-21	234	10	0	0	0.00	0	0.00	0	0.00
	27-Sep-21	136	20	7	0	0.00	5	3.68	1	0.74
	18-Oct-21	111	93	51	1	0.90	36	32.43	14	12.61
**Total sum**		***1717***	***335***	***155***	***76***		***45***		***27***	
**Percentage of parasitoid species by host**					***49.03***		***29.03***		***17.42***	

The colors used in the table indicate the different sections of the table: the upper shaded cells correspond to the column headings, whereas the lower shaded cells indicate the summary of the data.

**Table 2:** Immature forms by leaf and parasitism rates, total parasitism (Pt) and effective parasitism (Pe) (mean ± standard deviation), by psyllid species within plots. Castile and Leon, 2019 to 2021.

Plot	Date	Immature Forms/Leaf			Parasitism Rates	
		Eggs	N1-N2	N3-N5	Pt (%)	Pe (%)
*Bactericera tremblayi*						
CC-2019	30-Sep-19	0.39 ± 1.46	1.1 ± 1.82	0.77 ± 1.48	30.81 ± 39.87	7.06 ± 8.24
	12-Nov-19	0.09 ± 0.47	0.07 ± 0.29	3.05 ± 3.79	23.66 ± 7.59	18.19 ± 4.79
I-2020	03-Sep-20	1.3 ± 2.91	1.37 ± 2	2.77 ± 2.74	39.18 ± 7.72	19.34 ± 8.85
	21-Sep-20	0.94 ± 2.31	1.67 ± 3.07	2.74 ± 3.03	73.03 ± 5.4	27.29 ± 16.66
	05-Oct-20	0.45 ± 1.77	1.07 ± 1.64	2.35 ± 2.62	60.24 ± 13.54	25.55 ± 15.98
CC1-2021	22-Sep-21	0.02 ± 0.13	0.29 ± 1.15	0.3 ± 0.79	2.85 ± 6.38	2.85 ± 6.38
CC2-2021	20-Sep-21	0.04 ± 0.23	0.02 ± 0.16	0.2 ± 0.79	0 ± 0	0 ± 0
	04-Oct-21	0.001 ± 0.09	0.74 ± 1.5	1.93 ± 4.62	1.59 ± 2.18	0.5 ± 1.13
	27-Oct-21	0.001 ± 0.09	0.35 ± 1.45	3.12 ± 4.31	31.64 ± 12.03	18.12 ± 12.56
*Bactericera trigonica*						
AC-2019	16-Sep-19	4.11 ± 4.27	1.71 ± 1.93	3.2 ± 2.96	39.77 ± 7.67	36.3 ± 6.96
CC-2019	15-Oct-19	8.77 ± 8.49	6.2 ± 6.25	4.42 ± 4.63	10.61 ± 5.96	10.61 ± 5.96
	02-Dec-19	0.41 ± 1.14	0.66 ± 1.25	3.08 ± 3.17	1.91 ± 1.37	0.69 ± 0.96
I-2020	14-Sep-20	13.27 ± 15.45	4.01 ± 5.28	1.51 ± 3.05	24.26 ± 14.33	9.52 ± 3.03
	28-Sep-20	2.85 ± 4.01	1.83 ± 2.94	1.36 ± 2.66	21.31 ± 7.91	4.63 ± 5.89
	13-Oct-20	1.37 ± 2.74	0.85 ± 1.43	1.11 ± 1.43	22.35 ± 7.45	5.13 ± 3.84
CC-2021	06-Sep-21	5.32 ± 4.32	1.18 ± 1.29	0.99 ± 1.12	1.93 ± 4.32	0.64 ± 1.44
I-2021	13-Sep-21	16.16 ± 11.67	8.1 ± 7.04	2.22 ± 2.96	4.94 ± 5.42	0 ± 0
	27-Sep-21	5.3 ± 4.12	4.78 ± 4.32	4.14 ± 4.14	4.1 ± 2.57	2.28 ± 2.36
	18-Oct-21	2.8 ± 3.87	1.51 ± 2.44	4.9 ± 4.34	18.5 ± 2.58	14.2 ± 3.37

## Data Availability

Data are contained within the article or Supplementary Materials.

## Supplementary Materials

The following supporting information can be downloaded at: https://www.mdpi.com/article/10.3390/insects17080848/s1. Table S1. Location, information and dates of surveys of leek and carrot plots during 2019, 2020 and 2021 in Castile and Leon (Spain). Table S2. Repository of voucher specimens of parasitoids isolated from *Bactericera trigonica* and *B. tremblayi* collected in carrot and leek fields in Castile and León (Spain), 2019–2021. Table S3. Pairwise genetic distances between the parasitoid specimens obtained in this study and the closest reference sequences retrieved from GenBank and BOLD.
