# Supplementary material for: Parasitoid Diversity and Biological Control of Bactericera tremblayi and Bactericera trigonica (Hemiptera: Psylloidea) in Horticultural Crops in Spain
Source: Insects. 2026 Aug 14;17(8):848. doi: 10.3390/insects17080848 (PMC13513076; doi:10.3390/insects17080848)
Supplement: Supplementary file 1 [file insects-17-00848-s001.zip › Supplementary material_Table S3.pdf]

Supplementary material

Table S3. Pairwise genetic distances between the parasitoid specimens obtained in this study and the closest reference sequences retrieved from GenBank and BOLD.

	Tamarixia pronomus (PV132258) (*)	*Tamarixia tremblayi* (PV132259) (*)	*Syrphophagus taeniatus* (PV132260) (*)	*Syrphophagus taeniatus* (PV132261) (*)
Tamarixia upis (BCHYM14301-16)	0.0769	0.0841		
Tamarixia dryi (MK293954.1)	0.0951	0.1007		
Tamarixia radiata (GBMIN74767-17)	0.0989	0.0990		
Tamarixia pronomus (GBDTA12171-21)	0.0165	0.0574		
Tamarixia pronomus (PV132258) (*)		0.0644		
Psyllaephagus pilosus (NZHYM109-10)	0.2344	0.2257	0.2298	0.2298
Syrphophagus aphidivorus (ZFMK-TIS-2625907)	0.2569	0.2570	0.1740	0.1740
Syrphophagus sp. (GMPTJ4074-23)			0.1558	0.1558
