# Supplementary material for: Parasitoid Diversity and Biological Control of Bactericera tremblayi and Bactericera trigonica (Hemiptera: Psylloidea) in Horticultural Crops in Spain
Source: Insects. 2026 Aug 14;17(8):848. doi: 10.3390/insects17080848 (PMC13513076; doi:10.3390/insects17080848)
Supplement: Supplementary file 1 [file insects-17-00848-s001.zip › Supplementary material_Table S1.pdf]

Supplementary material

Table S1. Location, information and dates of surveys of leek and carrot plots during 2019, 2020 and 2021 in Castile and Leon (Spain).

Crop	Locality/Plot	Latitude	Longitude	Date of survey 1	Date of survey 2	Date of survey 3
leek	Campo de Cuéllar/CC-2019	41°18' 34.75''	4°21' 29.87''	30/09/2019	12/11/2019	
leek	Íscar/I-2020	41°20' 17.38''	4°31' 32.96''	03/09/2020	21/09/2020	05/10/2020
leek	Campo de Cuéllar/ CC1-2021	41°18' 32.99''	4°21' 11.11''	22/09/2021		
leek	Campo de Cuéllar/CC2-2021	41°18' 30.09''	4°21' 48.17''	20/09/2021	04/10/2021	27/10/2021
carrot	Arroyo de Cuéllar/AC-2019	41°21' 25.95''	4°20' 14.53''	16/09/2019		
carrot	Campo de Cuéllar/CC-2019	41°18' 40.86''	4°21' 29.03''	15/10/2019	02/12/2019	
carrot	Íscar/I-2020	41°20' 22.03''	4°31' 36.93''	14/09/2020	28/09/2020	13/10/2020
carrot	Campo de Cuéllar/CC-2021	41°19' 15.91''	4°21' 59.54''	06/09/2021		
carrot	Íscar/I-2021	41°20' 15.54''	4°31' 30.91''	13/09/2021	27/09/2021	18/10/2021
