# Supplementary material for: Parasitoid Diversity and Biological Control of Bactericera tremblayi and Bactericera trigonica (Hemiptera: Psylloidea) in Horticultural Crops in Spain
Source: Insects. 2026 Aug 14;17(8):848. doi: 10.3390/insects17080848 (PMC13513076; doi:10.3390/insects17080848)
Supplement: Supplementary file 1 [file insects-17-00848-s001.zip › Supplementary material_Table S2.pdf]

Supplementary material

Table S2. Repository of voucher specimens of parasitoids isolated from *Bactericera trigonica* and *B. tremblayi* collected in carrot and leek fields in Castile and León (Spain), 2019–2021.

	Lucian Fusu Collection, Al. I. Cuza University of Iaşi, Romania	*Lab. ITACyL*
Tamarixia pronomus		*130*
Tamarixia tremblayi		*72*
Syrphophagus herbidus	*10*	*67*
Syrphophagus cf. taeniatus	*4*	
Cheiloneurus	*1*	
Encyrtidae sp.	*1*	
Tamarixia sp.		*6*
